# GrantCheck—an AI Solution for Guiding Grant Language to New Policy Requirements: Development Study

**DOI:** 10.2196/79038

**Published:** 2025-11-27

**Authors:** Qiming Shi, Asil Oztekin, George Matthew, Jeffrey Bortle, Hayden Jenkins, Steven (Koon) Wong, Paul Langlois, Anaheed Zaki, Brian Coleman, Katherine Luzuriaga, Adrian H Zai

**Affiliations:** 1 Center for Clinical and Translational Science UMass Chan Medical School Worcester, MA United States; 2 Manning School of Business UMass Lowell Lowell, MA United States; 3 Information Technology UMass Chan Medical School Worcester, MA United States; 4 Department of Population and Quantitative Health Sciences UMass Chan Medical School Worcester, MA United States

**Keywords:** artificial intelligence, natural language processing, large language model, user-computer interface, grant applications, research compliance, usability evaluation

## Abstract

**Background:**

Academic institutions face increasing challenges in grant writing due to evolving federal and state policies that restrict the use of specific language. Manual review processes are labor-intensive and may delay submissions, highlighting the need for scalable, secure solutions that ensure compliance without compromising scientific integrity.

**Objective:**

This study aimed to develop a secure, artificial intelligence–powered tool that assists researchers in writing grants consistent with evolving state and federal policy requirements.

**Methods:**

GrantCheck (University of Massachusetts Chan Medical School) was built on a private Amazon Web Services virtual private cloud, integrating a rule-based natural language processing engine with large language models accessed via Amazon Bedrock. A hybrid pipeline detects flagged terms and generates alternative phrasing, with validation steps to prevent hallucinations. A secure web-based front end enables document upload and report retrieval. Usability was assessed using the System Usability Scale.

**Results:**

GrantCheck achieved high performance in detecting and recommending alternatives for sensitive terms, with a precision of 1.00, recall of 0.73, and an *F*_1_-score of 0.84—outperforming general-purpose models including GPT-4o (OpenAI; *F*_1_=0.43), Deepseek R1 (High-Flyer; *F*_1_=0.40), Llama 3.1 (Meta AI; *F*_1_=0.27), Gemini 2.5 Flash (Google; *F*_1_=0.58), and even Gemini 2.5 Pro (Google; *F*_1_=0.72). Usability testing among 25 faculty and staff yielded a mean System Usability Scale score of 85.9 (SD 13.4), indicating high user satisfaction and strong workflow integration.

**Conclusions:**

GrantCheck demonstrates the feasibility of deploying institutionally hosted, artificial intelligence–driven systems to support compliant and researcher-friendly grant writing. Beyond administrative efficiency, such systems can indirectly safeguard public health research continuity by minimizing grant delays and funding losses caused by language-related policy changes. By maintaining compliance without suppressing scientific rigor or inclusivity, GrantCheck helps protect the pipeline of research that advances biomedical discovery, health equity, and patient outcomes. This capability is particularly relevant for proposals in sensitive domains—such as social determinants of health, behavioral medicine, and community-based research—that are most vulnerable to evolving policy restrictions. As a proof-of-concept development study, our implementation is tailored to one institution’s policy environment and security infrastructure, and findings should be interpreted as preliminary rather than universally generalizable.

## Introduction

Recent shifts in federal and state policies have imposed restrictions on certain terminology in grant applications, creating new challenges for researchers during proposal development [1-3]. Terms related to concepts such as diversity, gender identity, and climate change [4] have, in some cases, been informally “flagged” or discouraged in funding submissions. This evolving policy environment has introduced uncertainty and increased administrative workload: grant offices must now invest additional time in language screening, and investigators risk delays or rejection if proposals contain politically sensitive terms [5-8]. These pressures highlight an urgent need for tools that enable institutions to ensure compliance with language requirements while preserving the clarity and integrity of scientific communication. These evolving requirements have far-reaching implications for biomedical and public health research. Policy-related grant delays or rejections can interrupt ongoing studies in cancer prevention, infectious disease surveillance, or chronic disease management, leading to gaps in data continuity and slower translation of findings into clinical or population-level benefit. Furthermore, proposals focused on health equity or community engagement are disproportionately affected by language restrictions, risking underrepresentation of vulnerable populations in federally funded research. Addressing these challenges is therefore not merely an administrative priority but a health systems imperative—ensuring compliant communication safeguards the research pipeline that underpins patient care innovation, disease prevention, and equitable health outcomes.

To address this challenge, we pose the following research question: Can a domain-specific artificial intelligence (AI) system automatically detect policy-restricted language in grant documents and assist in revising it to meet new requirements, while maintaining the integrity of the scientific content? Our objective is to develop and evaluate a solution that achieves high accuracy in identifying noncompliant phrases and providing appropriate alternatives. Evaluation metrics include precision and recall in detecting flagged terms, as well as user-centered measures such as the System Usability Scale (SUS) to assess the acceptability and practical utility of the tool’s recommendations.

A variety of writing-support technologies have emerged to help researchers navigate increasingly complex requirements. General-purpose AI writing assistants, such as Grammarly (Superhuman Platform Inc) [9] and ChatGPT (OpenAI) [10], can refine grammar and style but are not designed to enforce domain-specific policies. In response, several grant-specific tools have been proposed, and multiple peer-reviewed studies have examined large language models (LLMs) directly in the context of grant writing. Seckel et al [11] provided structured guidelines for ethical and effective LLM use in proposal preparation, highlighting their utility for literature summarization and clarity improvements while cautioning against overreliance and plagiarism risks. Godwin et al [12] reported that a guided generative AI tool for National Institutes of Health grant drafting yielded substantial time savings and enhanced workflow efficiency. Meyer et al [13] observed that LLMs can accelerate early drafting by overcoming writer’s block, although human revision is essential to ensure specificity and policy compliance.

In addition, LLMs have been leveraged to streamline documentation and policy adherence. Fukataki et al [14] demonstrated that GPT-4 (OpenAI), when combined with tailored prompts, could accurately extract ethically relevant information from research protocols for Institutional Review Board (IRB) prereview, achieving high reproducibility and alignment with human reviewer assessments. Similarly, Heilmeyer et al [15] showed that domain-tuned open LLMs deployed in a privacy-preserving local environment produced high-quality clinical documentation, with 93% of outputs requiring minimal or no edits before integration into medical records. LLMs have also been shown to enhance general scientific and medical writing. Huang and Tan [16] reported that ChatGPT can improve efficiency and writing quality in scientific review article preparation, particularly for expanding content and refining style.

Collectively, these studies demonstrate the potential for LLMs to support domain-specific writing and compliance-related document preparation. However, to our knowledge, there is no previous study that addresses compliance requirements in grant language while simultaneously delivering an institutionally hosted solution designed to mitigate confidentiality risks.

Building on this body of work, GrantCheck (University of Massachusetts Chan Medical School) introduces an institutionally hosted, hybrid AI system that combines rule-based natural language processing (NLP) with LLM-based rephrasing to support compliant grant writing. Unlike previous tools, GrantCheck explicitly targets policy-sensitive term detection under evolving funding guidelines and provides secure, context-appropriate alternatives within a private cloud environment—addressing both compliance requirements and confidentiality concerns. This line of innovation carries important implications for the broader research and health ecosystem. As grant restrictions increasingly affect studies on population health, behavioral science, and health equity, tools like GrantCheck can help preserve scientific progress in these sensitive domains by ensuring compliant communication rather than topic avoidance. In doing so, GrantCheck not only streamlines institutional compliance but also supports the continuity of critical health research addressing disparities, chronic disease prevention, and emerging public health priorities.

## Methods

### Overview

This development study was structured as a two-part evaluation: (1) performance testing of GrantCheck using a gold-standard annotated dataset, and (2) usability testing with faculty and research staff. The intervention was delivered via a secure web-based application where participants uploaded grant documents, which were processed by the hybrid NLP–LLM pipeline. Output reports were automatically generated and returned to users through the same interface.

### Ethical Considerations

This study was reviewed by the IRB of UMass Chan Medical School (STUDY00002481) and was determined not to constitute research involving human participants under Department of Health and Human Services and Food and Drug Administration regulations. Consequently, formal IRB oversight and informed consent were not required. Usability testing was conducted with faculty and staff members who voluntarily participated in the SUS survey. Participation was anonymous, and no identifiable personal data were collected. All procedures complied with institutional policy, the principles outlined in the Declaration of Helsinki, *Journal of Medical Internet Research*’s ethical requirements for usability studies, and Committee on Publication Ethics guidelines for responsible research conduct. No compensation was provided to participants.

### Infrastructure Layer

To support the secure and scalable deployment of GrantCheck, the system was implemented within Amazon Web Services (AWS) Cloud, ensuring strict adherence to institutional data security and privacy standards. This backend infrastructure supports the integration of advanced LLMs through Amazon Bedrock [17] while maintaining a fully secured environment. Microsoft Single Sign-On (SSO) authentication restricts front-end access to authorized users.

GrantCheck’s back end runs in a private, encrypted AWS virtual private cloud (VPC) without direct exposure to the public internet. The infrastructure includes Amazon Bedrock for LLM inference (Amazon Nova Pro and Meta Llama 3.1 70B Instruct), Identity and Access Management–controlled AWS Lambda serverless function hosting the processing engine and orchestration logic, handling data in memory without persistent storage. While user uploads are temporarily stored in Amazon S3 to support processing, a lifecycle rule automatically deletes these files after 1 day, ensuring minimal data retention. All data in transit are protected using Transport Layer Security 1.3 encryption, and temporary cryptographic keys are managed via AWS Key Management Service.

**Figure 1:**
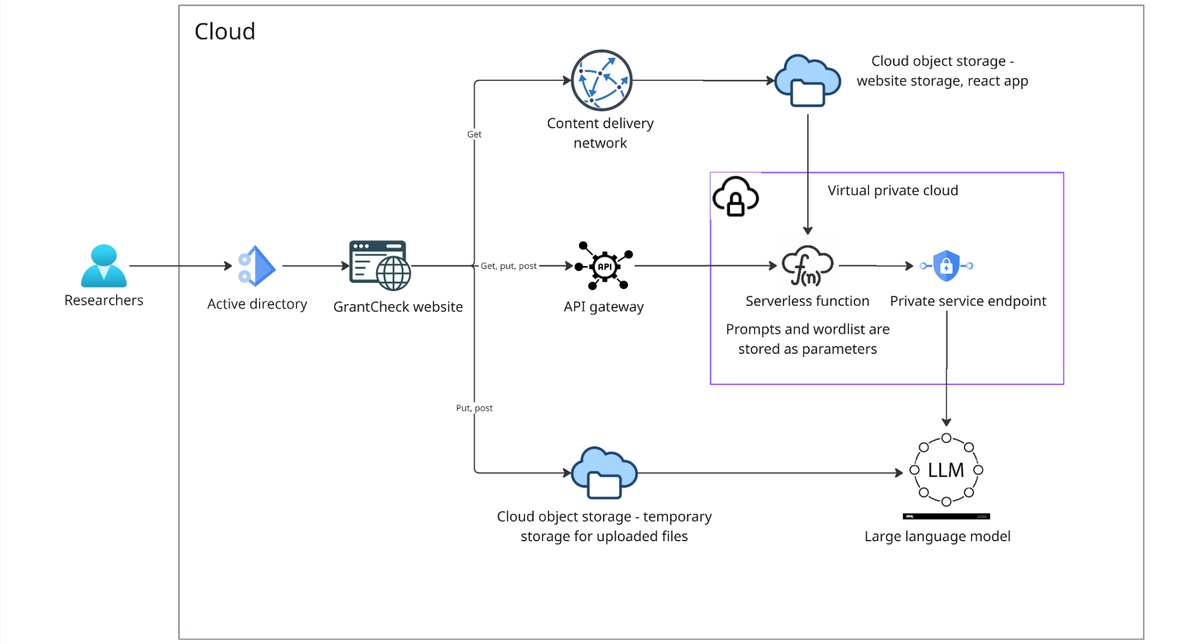
Cloud infrastructure. API: application programming interface.

Access to the tool is gated through SSO, with audit logging implemented across application and network layers to support traceability. The AWS infrastructure diagram (Figure 1) provides an overview of how the components interact within the secure environment.

Figure 1 depicts the cloud infrastructure supporting the application, including authentication, frontend, backend processing, and LLM orchestration through Cloud, all within a private, encrypted VPC environment.

### Security Measures

GrantCheck is currently deployed in a preproduction environment within an AWS VPC and was therefore not included in the most recent institutional third-party penetration testing cycle. Upon production release, the system will be incorporated into our institution’s routine penetration testing process, which is performed by an external vendor under the direction of the Information Security Office. The application’s configuration adheres to institutional security controls aligned with established standards such as the National Institute of Standards and Technology and the Center for Internet Security benchmarks.

Per-user data isolation is enforced through AWS Amplify’s built-in storage authorization, ensuring that files are accessible only to their respective owners. Session token expiration is managed via Amazon Cognito, with refresh tokens expiring after 1 day and both access and ID tokens expiring after 1 hour. Although formal prompt injection resistance testing has not yet been conducted, it is planned as part of the security validation process before production deployment. These measures, combined with the isolated VPC environment and session-based memory clearing, help safeguard user data and maintain compliance with institutional security requirements.

### Backend Processing Pipeline

A hybrid pipeline was implemented to improve the precision and reliability of sensitive language detection. It combines rule-based NLP with LLM inference. This layered approach mitigates the limitations of LLM-only methods, especially concerning hallucination, token leakage, and contextual ambiguity.

The initial processing stage uses a custom NLP module built with Python’s regular expression (regex) library [18]. We developed a curated dictionary of 238 sensitive terms and phrases for language identification, with the majority derived from Appendix B of the Senate guidelines [19]. This list was expanded through multiple sources and underwent thorough review and approval by the school’s faculty council and executive committee, ensuring that it extended beyond terminology alone to reflect emerging areas of policy sensitivity. For example, terms such as “vaccine hesitancy” and “vaccine uptake” were included, given their recent association with grant cancellations [20]. To ensure the dictionary remains up-to-date and accurate, a feedback system is in place where researchers can suggest additions, which undergo thorough review before potential integration into the existing list. Terms are annotated with contextual rules to distinguish benign from problematic uses.

Regex patterns were programmatically generated to ensure consistent and reproducible matching across the sensitive-term dictionary. Patterns were designed with word boundaries to capture only whole words, and case-insensitive matching was applied to avoid missing capitalized variants. For multiword terms, a single fixed whitespace was used to ensure precise detection.

When a document is uploaded, the engine scans its content to identify sensitive terms using regex, extracts the surrounding sentence for context, and generates a structured table listing each term along with its corresponding sentence. The structured table is passed to an LLM via Amazon Bedrock in the second stage to make an alternative term for replacement. Model parameters are tuned with a temperature of 0.7, a top-p setting of 0.9, and a maximum token count of 5000. These settings are version-controlled to ensure consistent inference.

**Figure 2:**
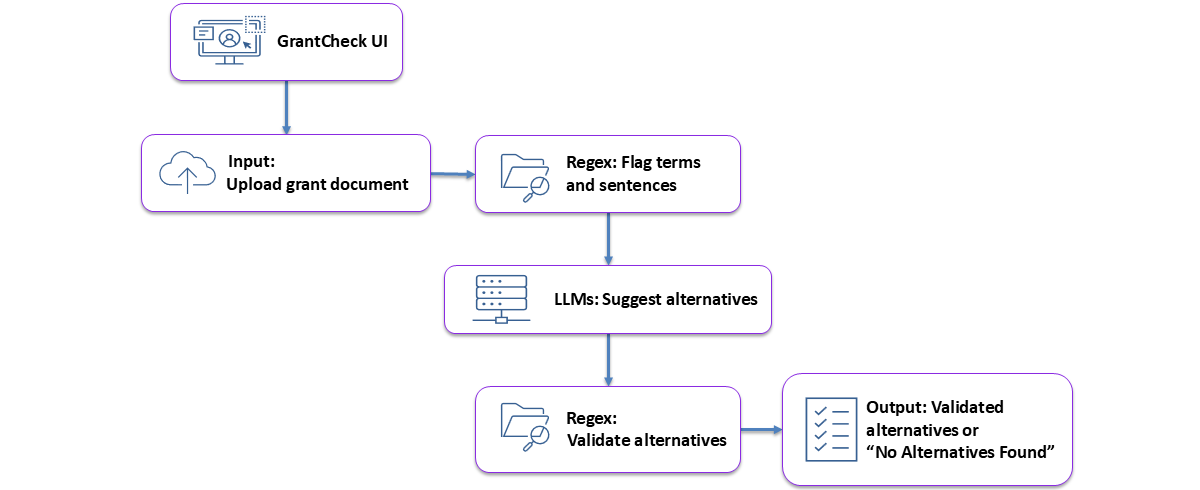
End-to-end pipeline for regex-guided sensitive term replacement via large language model. LLM: large language model; UI: user interface.

Following LLM inference, the regex engine re-evaluates the proposed alternative text. If any flagged sensitive term is found in the LLM’s output, the replacement is suppressed and replaced with a standard message: “No safe alternative found.” This safeguard was added to address residual cases of hallucination or subtle term reintroduction. The end-to-end pipeline for this process is illustrated in Figure 2.

Figure 2 illustrates the document flow through the application, beginning with document upload, followed by sensitive term detection, LLM-based suggestion generation, post-processing validation, and finalized output.

### Front End Interface

**Figure 3:**
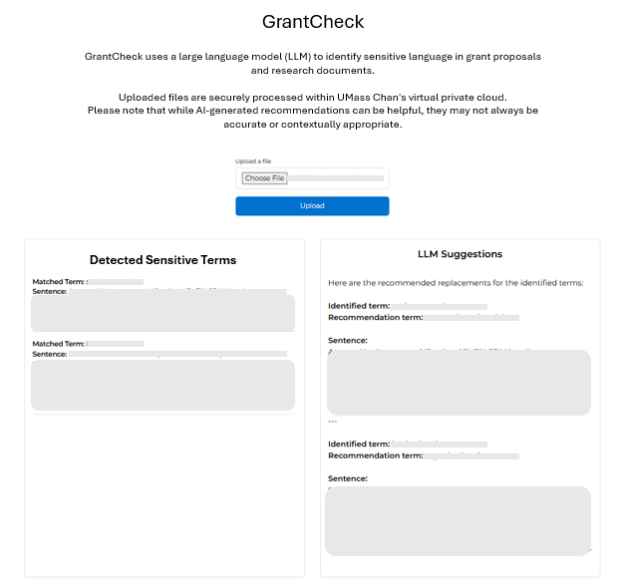
GrantCheck user interface.

A lightweight, web-based front end (Figure 3) was developed to provide a secure, user-friendly interface for document upload and report retrieval. This interface was custom-built to comply with institutional security policies. The front end enables users to upload grant documents, which are transmitted securely to the backend processing pipeline. Once the analysis is complete, the user is presented with a report summarizing flagged sensitive terms and their suggested alternatives. Access to the application is limited to authenticated users through Microsoft SSO. The front end does not store any document content; data are stored in S3 for up to 1 day.

Figure 3 illustrates the front end interface presented to users during the beta testing phase. The interface includes a brief description of GrantCheck’s function, a file upload field, and an action button for submission. Results are presented in 2 sections—one listing the detected sensitive terms and another showing suggestions generated by LLM. Documents are processed within the secure VPC, and users are reminded that suggestions are AI-generated and may not always be accurate.

### Performance Evaluation

To assess GrantCheck’s effectiveness in identifying sensitive language, we conducted a comparative evaluation using a gold standard dataset derived from manually annotated grant proposals. In total, 5 documents were manually annotated by 2 annotators with extensive experience in grant writing and compliance review. Each document was reviewed against the institution’s sensitive-term dictionary. True positives, false positives, and false negatives were identified for precision, sensitivity, and specificity calculation. Because identification of terms from the curated sensitive word list is a relatively deterministic task with minimal subjective interpretation, each document was annotated once by a single annotator rather than independently by multiple annotators. We acknowledge that this choice precluded formal assessment of interannotator agreement and represents a limitation that future validation studies should address through double annotation and calculation of interrater reliability metrics such as Cohen κ. The set included both active grant applications under development at our institution and scholarly articles with a strong emphasis on Diversity, Equity, and Inclusion–related principles, selected to test the system’s maximum detection capability. Because identifying terms from the curated sensitive word list is a straightforward task with minimal subjective interpretation, each document was annotated once by a single annotator, rather than having both annotators independently annotate the same document to calculate interannotator agreement. In total, 2 system configurations were tested: version 1, which used a general-purpose LLM with prompts applied to the full document, and version 2 (GrantCheck), which implemented a hybrid pipeline combining rule-based regex detection with targeted LLM inference. For each model, we constructed contingency tables of true positives, false positives, true negatives, and false negatives, and calculated precision, sensitivity (recall), and specificity [21,22]. Furthermore, 95% CIs for these metrics were estimated via 5000 bootstrap resamples [23], and the McNemar exact test [24] was used to assess whether observed differences in error rates between GrantCheck and baseline models were statistically significant (*P*<.05). This framework provided a comprehensive diagnostic evaluation that captured both performance accuracy and error patterns.

### Usability Testing

A total of 25 participants were recruited for the SUS survey [25] following statistical guidance from Sauro and Lewis [26], which indicates that, given the typical standard deviation of SUS scores (approximately 12.5), this sample size yields a 95% CI of approximately ±5 points—an accepted precision level for usability benchmarking. This number balances statistical rigor with resource efficiency. Eligible participants were faculty and research staff with recent grant-writing experience, representing the intended end user group for GrantCheck. A convenience sample of 30 faculty and staff with recent grant-writing experience was invited via institutional email, and 25 participated (response rate 83%). All participants completed the SUS in full, with no partial responses. The cohort included 20 principal investigators and 5 scientific writers from diverse departments, including Population & Quantitative Health Sciences, Pulmonary Medicine, the Center for Clinical and Translational Science, Genetics & Cellular Medicine, Cardiovascular Medicine, Molecular, Cell & Cancer Biology, Medicine, Microbiology, Health Systems Science, Emergency Medicine, Pediatrics, Surgery, Family Medicine & Community Health, and Neurobiology.

During the beta testing phase, participants completed the SUS survey (Multimedia Appendix 1), which included 10 structured questions scored on a 5-point Likert scale to evaluate user experience, interface design, and overall satisfaction. An additional open-ended question invited recommendations and suggestions for improvement. Survey responses were collected anonymously, and participation was voluntary. All usability data were reviewed to guide iterative refinements to the user interface and workflow integration. This activity was determined not to constitute human participants research and did not require IRB oversight under institutional policy.

To compute the SUS score, responses to positively worded items (items 1, 3, 5, 7, and 9) were adjusted by subtracting 1 from the raw score. For negatively worded items (items 2, 4, 6, 8, and 10), responses were reverse scored by subtracting the raw score from 5. Each adjusted item score ranged from 0 to 4, and the sum of all 10 adjusted item scores was multiplied by 2.5 to yield a final SUS score ranging from 0 to 100. Higher scores indicate better usability. Scores ≥68 were interpreted as above average. Descriptive statistics (mean, SD, minimum, maximum, and median) were reported for overall SUS results, and open-text feedback was reviewed qualitatively to identify themes related to feasibility and acceptability.

### Security and Data Governance

Our Information Security Office reviewed and approved the backend infrastructure design. User uploads are temporarily stored in S3 and automatically deleted after one day through a defined lifecycle rule. Once analysis is completed, the session is terminated, and all memory is cleared within 1 day. To mitigate risks of prompt injection and unintentional data retention, users do not interact with the LLM directly, and the model end points are isolated from internet-facing services. Security audits and threat reviews are conducted quarterly to ensure ongoing compliance. In addition, web application vulnerability scans are performed before each deployment to identify and mitigate potential risks before changes go live.

### Plan-Do-Study-Act Cycle

We continuously incorporated user feedback into the development process, using it to refine and enhance the tool through iterative updates.

## Results

Across the annotated evaluation set, GrantCheck achieved the strongest overall diagnostic performance (Table 1). It correctly identified 16 of 22 policy-sensitive terms (true positive) while producing no false positives, yielding perfect precision and specificity (both 100%) and a sensitivity of 73% (95% CI 56%-87%). By contrast, ChatGPT-4o (OpenAI), Deepseek R1 (High-Flyer), and Llama 3.1 70B (Meta AI) showed substantially lower sensitivities (41%, 32%, and 18%, respectively) coupled with false positives that reduced specificity. Gemini 2.5 Flash (Google) demonstrated moderate performance (sensitivity 50% and precision 69%), while Gemini 2.5 Pro approached GrantCheck in precision (93%) and specificity (95%) but exhibited lower sensitivity (59%).

Exact McNemar tests confirmed that GrantCheck’s improvements over ChatGPT-4o, Deepseek R1, and Llama 3.1 were statistically significant (*P*<.05), while the difference versus Gemini 2.5 Flash was borderline (*P*=.09). The comparison with Gemini 2.5 Pro was not statistically significant (*P*=.42), reflecting its relatively strong performance on this dataset. These findings highlight GrantCheck’s ability to maximize compliance safety by avoiding false positives, while retaining higher sensitivity than competing general-purpose LLMs.

**Table 1:** Comparative diagnostic performance of GrantCheck and baseline models in detecting policy-sensitive terms.

Model	TP^a^	FP^b^	TN^c^	FN^d^	Precision (95% CI)	Sensitivity (95% CI)	Specificity (95% CI)	McNemar vs Amandla (*P* value)
GrantCheck (Amandla)	16	0	20	6	1.00 (0.96-1.00)	0.73 (0.56-0.87)	1.00 (0.95-1.00)	—^e^
ChatGPT-4o	9	11	9	13	0.45 (0.26-0.65)	0.41 (0.23-0.59)	0.45 (0.25-0.65)	.04
Deepseek R1	7	6	14	15	0.54 (0.29-0.77)	0.32 (0.14-0.50)	0.70 (0.50-0.85)	.01
Llama 3.1 70B	4	4	16	18	0.50 (0.20-0.80)	0.18 (0.04-0.36)	0.80 (0.61-0.95)	.001
Gemini 2.5 Flash	11	5	15	11	0.69 (0.44-0.89)	0.50 (0.31-0.69)	0.75 (0.56-0.90)	.09
Gemini 2.5 Pro	13	1	19	9	0.93 (0.71-1.00)	0.59 (0.40-0.77)	0.95 (0.82-1.00)	.42

^a^TP: true positive—cases correctly identified as positive.

^b^FP: false positive—cases incorrectly identified as positive.

^c^TN: true negative—cases correctly identified as negative.

^d^FN: false negative—cases incorrectly identified as negative.

^e^Not available.

As illustrated in Table 2, the mean final SUS score across participants was 85.90 (SD 13.44), suggesting above-average usability. The score distribution ranged from 55 to 100, with a median of 92.5 (IQR 80.0-98.0), indicating that most participants found the system highly usable. Individual item means ranged from 3.08 to 3.80 on a scale of 0 to 4. Given that a score above 2 is above the midpoint, this indicates generally positive responses across all items. The highest agreement was observed on the need for minimal technical support and ease of learning, while slightly lower scores were noted for perceived consistency and user confidence. Overall, the item-level averages suggest the application performs well in supporting usability.

**Table 2:** Usability survey results.

SUS^a^ item	Mean (SD)	Median (IQR)
I think that I would like to use this system frequently	3.16 (0.94)	3.00 (2.00-4.00)
I found the system unnecessarily complex	3.52 (1.16)	4.00 (3.00-4.00)
I thought the system was easy to use	3.64 (0.64)	4.00 (3.00-4.00)
I think that I would need the support of a technical person to be able to use this system	3.76 (0.83)	4.00 (3.00-4.00)
I found the various functions in this system were well integrated	3.08 (0.95)	3.00 (2.00-4.00)
I thought there was too much inconsistency in this system	3.08 (1.15)	4.00 (3.00-4.00)
I would imagine that most people would learn to use this system very quickly	3.80 (0.50)	4.00 (3.00-4.00)
I found the system very cumbersome to use	3.68 (0.75)	4.00 (3.00-4.00)
I felt very confident using the system	3.28 (0.89)	4.00 (2.00-4.00)
I needed to learn a lot of things before I could get going with this system	3.36 (1.19)	4.00 (2.00-4.00)
Final SUS score (0-100)	85.90 (13.44)	92.50 (80.00-98.00)

^a^SUS: System Usability Scale.

In addition to the SUS scores, open-ended feedback revealed four recurring themes: (1) export and print functionality, with users requesting the ability to export flagged results into Microsoft Word documents and print reports; (2) clarity and guidance on how to interpret and act upon flagged content, highlighting the need to pair technical recommendations with integrated educational support; (3) accuracy and utility of suggestions, as some replacements such as “women’s health” to “midwifery health” were viewed as unhelpful or overly rigid, whereas substitutions such as replacing “diversity” with “broad representation” and “equity” with “fair access” were considered helpful and appropriate; and (4) highlighting and replacement functionality, with requests for in-document highlighting and inline replacement options rather than a separate spotter report.

## Discussion

### Design and Features

The development of GrantCheck reflects a practical response to a rapidly evolving policy landscape in academic and research institutions. While this study demonstrates feasibility, it should be understood as a proof-of-concept development study tailored to one institution’s curated dictionary, policy interpretations, and security requirements. As such, the findings provide preliminary evidence of feasibility but are not intended to suggest universal generalizability. As funding agencies and state governments implement new language-related restrictions, institutions face significant challenges in aligning grant narratives with compliance requirements while maintaining the scientific integrity of their proposals. GrantCheck addresses this emerging need by providing a secure, institutionally compliant solution that assists users in identifying and revising potentially sensitive terminology using a hybrid regex–LLM pipeline.

Initially, we experimented with GPT-4o using few-shot prompting, which yielded promising results but revealed limitations in both overcalling and undercalling sensitive terms. To address these issues, we developed GrantCheck, a hybrid NLP pipeline that integrates rule-based term detection with LLM inference. This approach balances the precision of deterministic methods with the contextual sensitivity of generative AI. A postprocessing validation step further enhances system reliability by mitigating hallucinations and preventing the inadvertent reintroduction of sensitive language. These results should be interpreted with caution, as they are based on a small, annotated dataset and a single-institution user group. Our evaluation shows that GrantCheck consistently achieved perfect precision and specificity, with no false positives across the test set. Our reported performance metrics should be interpreted as preliminary findings based on a small, annotated dataset, designed to demonstrate feasibility rather than to provide definitive benchmarks. This outcome was confirmed through gold-standard annotation and reflects the tool’s conservative design, which combines regex-based deterministic detection with a safeguard that suppresses any LLM outputs reintroducing flagged terms. This feature is critical in the grant compliance context, as overcalling benign terms could unnecessarily disrupt proposal preparation, introduce avoidable edits, and risk misinterpretation by reviewers. Its sensitivity of 73% indicates that most, but not all, sensitive terms are detected. By contrast, general-purpose LLMs (eg, ChatGPT-4o, Deepseek R1, and Llama 3.1) showed significantly weaker performance across both sensitivity and specificity, while Gemini 2.5 Pro emerged as the strongest baseline competitor with high precision (93%) and specificity (95%) but lower sensitivity (59%). Reporting 95% CIs and conducting McNemar exact tests confirmed that GrantCheck’s improvements over ChatGPT-4o, Deepseek R1, and Llama 3.1 were statistically significant, while differences with Gemini 2.5 Pro were not, underscoring its relative strength. The error analysis provided further insight into system behavior. False negatives in GrantCheck were primarily attributable to information overflow when large numbers of flagged segments exceeded the LLM’s optimal processing capacity, causing some terms to be dropped from the suggestion stage. This issue is discussed in more detail later in the manuscript as a limitation of long-context inference. By contrast, baseline models produced false negatives for different reasons, most often missing sensitive terms in less common formulations, and also generated false positives by overflagging benign technical usage. These patterns highlight that GrantCheck’s conservative design intentionally prioritizes avoiding false positives—an essential feature in compliance contexts where benign terms should not be mistakenly flagged.

From an infrastructure perspective, the deployment of GrantCheck within a VPC environment demonstrates the feasibility of securely operationalizing LLM-based tools for institutional use. This architecture, which isolates all data processing from public end points and enforces session-based memory clearing, is a model for responsible AI deployment in academic settings. From a cost-efficiency perspective, integrating a regex preprocessing step allows the system to extract and summarize only relevant segments before invoking the LLM. This significantly reduces token consumption by avoiding full-document processing, thereby lowering computational costs without compromising output quality. For example, in a grant containing a high density of sensitive terms, a typical run might involve approximately 3000 input tokens and 500 output tokens. On Amazon Nova Pro pricing, this equates to approximately US $0.036 per request. Even under an upper-bound assumption of 30 users each running the tool once per day, the estimated daily cost would be US $1.08, underscoring that real-world institutional costs are expected to remain very modest relative to the value of preventing grant rejections.

The usability testing results indicate that tools like GrantCheck can be seamlessly integrated into research workflows without burdening end users. Using the SUS enabled standardized assessment, while open-ended feedback informed iterative improvements. Early users helped uncover key limitations—specifically, overcalling and undercalling issues without a regex layer. In response, we implemented a regex-based component to extract identified sensitive terms and their corresponding sentence contexts. These outputs are then passed to the LLM, which generates recommended alternatives. This 2-step approach enhances reliability by narrowing the input scope, mitigating the risk of the LLM content and coherence loss when processing longer text, a known limitation of LLMs. Several users suggested providing a detailed report of the regex-identified terms, including their locations in the source text. To address this, we added a dual-pane display: one window shows each term and its sentence context, while the other presents the LLM’s recommended alternatives. One researcher noted uncertainty about whether the term list was exhaustive. By surfacing all regex-identified terms explicitly, the tool builds confidence in its comprehensiveness.

Within a month of its launch, the tool received 128 access requests from researchers across a wide range of departments. This early uptake highlights both the tool’s perceived value and the broad relevance across scientific disciplines. The broad user base also suggests strong institutional demand for automated support in navigating shifting grant policies.

### Limitations

Several limitations should be noted. GrantCheck was developed and evaluated within a single institution using a locally curated dictionary and infrastructure, so its broader applicability should be interpreted with caution. The findings are best understood as an early proof of concept, with broader impact requiring replication and adaptation at other institutions. In addition, our evaluation relied on a small, annotated dataset (n=5) and a single-institution user group (n=25). While this provided adequate precision for pilot usability assessment, the small sample limits statistical power and generalizability. We also did not conduct interannotator agreement testing, which could have quantified the reliability of manual annotations. Future work will therefore involve larger, multi-institutional datasets and double annotation with formal reliability statistics (eg, Cohen κ) to provide more robust performance benchmarks. The capabilities of the available LLMs on Amazon Bedrock constrain the system’s performance. Furthermore, while the regular expression engine reduces false positives and provides deterministic guardrails, it may miss nuanced or contextually embedded terms. The reliance on a curated dictionary also means updates must be manually maintained to align with policy changes.

GrantCheck is designed to flag all terms included in the institution’s approved sensitive-term dictionary, regardless of surrounding context. This approach reflects current policy conditions in which the mere presence of certain terms—whether used in a benign or technical sense—can lead to administrative hold or outright rejection before scientific review. As such, contextual disambiguation was deliberately excluded to minimize the risk of underdetection. While this may result in flagging terms that are not problematic in some settings, it ensures comprehensive identification of language that could trigger compliance concerns under prevailing interpretations.

Another known limitation arises when a document contains a large number of sensitive terms, as demonstrated in our experiments. In such cases, the regex engine successfully identifies all instances and extracts their corresponding sentence contexts; however, when this volume of information is passed to the LLM for alternative language suggestions, the long input may exceed the model’s optimal processing capacity. As a result, the LLM may fail to generate recommendations for some flagged terms due to input overload. Our testing showed that, for Amazon Nova Pro, this effect typically emerges when the combined term-context segments exceed approximately 2500 words. This issue is less probable in proposals with few sensitive terms. This 2500-word limit now serves as a benchmark for future optimization strategies, such as batching flagged terms or staging recommendations, to maintain high completion rates even in proposals with a large number of sensitive terms. Furthermore, researchers can iteratively resubmit revised versions of the proposal after addressing some flagged terms, thereby reducing input length and improving LLM response accuracy in subsequent passes. Importantly, even if a term is omitted from the recommendation output, it still appears in the regex findings window, allowing researchers to manually locate and revise it as needed. This ensures that no term is entirely lost in the review process, even if the LLM fails to return a suggestion.

Another limitation of GrantCheck is that its current implementation is tailored to the specific technical and policy environment of our institution. Model choice is constrained by security approvals, which until recently permitted only Amazon Nova and Llama 3.1 within our private VPC; however, at the time of this publication, our institution has obtained approval for access to GPT-4o, which we anticipate will enhance coverage and contextual accuracy in future iterations. Policy-specific term lists are likewise institution-dependent: while our curated dictionary was expanded through faculty council review and local policy interpretations, its foundation is drawn primarily from the Senate report, and broader use would require adapting or rebuilding the list in accordance with that report and the regulatory landscape at other organizations. Security requirements also constrain portability, as our deployment relied on an isolated AWS VPC and institutionally mandated SSO, which may differ across sites. While these constraints limit immediate transferability, the underlying architecture is flexible and can be adapted by other institutions through integration of their own approved models, curated term dictionaries, and security controls. Although GrantCheck was developed within the technical and policy context of a single US academic medical center, the underlying architecture is transferable. Other institutions could adapt the approach by substituting their own sensitive-term lists, approved LLMs, and security controls. Our findings, therefore, demonstrate feasibility but do not imply universal readiness.

A separate limitation involves access to more advanced LLMs. Due to our institution’s information security clearance regarding which LLMs we can use safely, the most powerful AI models accessible are Amazon Nova and Llama 3.1. These models provide a lower level of performance when compared with more advanced models such as GPT-4o. We believe that GrantCheck’s recommendation quality—both in terms of coverage and the appropriateness of suggested alternatives—will improve with access to more powerful models. To address this limitation and expand functional capabilities, our next stage of development will extend GrantCheck into a multiagent architecture. In this design, 1 subagent will retain the current role of detecting policy-sensitive terms, while a second subagent and its subagents will evaluate whether the grant proposal aligns with administrative and funding agency priorities. This second subagent will leverage tool-calling functions to provide targeted guidance on sections that may benefit from additional emphasis or improvement. A consolidating agent will then synthesize the outputs from these subagents into a unified, prioritized set of recommendations. This roadmap aims to address current limitations by expanding beyond term detection to offer richer, policy-aware feedback, while maintaining the security and compliance features of the existing system.

An additional limitation is that GrantCheck has not yet been formally benchmarked against the Coalition for Health AI [27] assurance framework, which emphasizes transparency, fairness, safety, and continuous monitoring in health-related AI applications. While current safeguards, such as VPC isolation, file deletion, and access controls, provide a strong technical foundation, future work will require mapping GrantCheck’s design and evaluation more explicitly to Coalition for Health AI principles to ensure alignment with emerging best practices in health care AI governance. Importantly, UMass Chan Medical School is home to the Massachusetts AI Assurance for Healthcare Laboratory [28], a nationally recognized initiative dedicated to advancing responsible and trustworthy AI in health care. Future iterations of GrantCheck will draw on this institutional expertise to strengthen assurance, benchmarking, and compliance with evolving governance standards. In addition, we plan to expand benchmarking and validation through multisite collaborations facilitated by the Massachusetts AI Assurance for Healthcare Laboratory at UMass Chan Medical School. These collaborations will enable the use of larger, more diverse document sets and external user groups, which are essential for establishing the external validity and generalizability of GrantCheck across different institutional contexts.

Currently, the tool identifies and suggests replacements for sensitive language, but future updates could also help researchers assess whether their proposals align with funding agency priorities. The tool’s underlying architecture is designed to be extensible, enabling future capabilities such as section prioritization, sentiment analysis, and policy-aware rewriting. Once licensing and infrastructure allow, expanding model options beyond Nova and Llama may further improve performance and adaptability.

One important practical note is that the DataFrame must be converted to a string before passing it to an LLM. Feeding a raw DataFrame directly to the LLM can result in loss of structural or contextual information, which is a limitation of how DataFrames are represented in memory. To preserve the data’s integrity and readability, it is recommended to use the .to_string() function to transform the data frame to text before submitting it to the model.

Despite these limitations, GrantCheck represents a step forward in operationalizing AI to support research compliance and integrity. Tools like GrantCheck can play a critical role in advancing scientific innovation by enabling researchers to adapt to shifting policies and funding agency priorities while preserving the meaning of their work.

Beyond technical performance, GrantCheck was developed with careful attention to ethical and governance considerations. The system operates solely in an advisory role, supporting researchers in identifying language that may warrant review under evolving administrative guidance. All suggestions are optional and remain under full user discretion, ensuring that authors maintain complete control and academic autonomy. The term dictionary and recommendation framework were developed through transparent institutional review and faculty consultation to uphold neutrality and accountability. The tool does not block, alter, or report content and operates entirely within a secure computing environment. To ensure that the system remains aligned with its advisory purpose, user feedback is actively collected and incorporated into regular updates. Because policies and interpretations evolve over time, GrantCheck is designed to be policy-agnostic and adaptable, allowing institutions to adjust its scope as guidance changes. In this way, the system promotes clarity, consistency, and efficiency in grant writing while safeguarding academic independence and avoiding any form of viewpoint enforcement or censorship.

From a health systems and translational research perspective, the implications of GrantCheck extend well beyond administrative compliance. Language restrictions and shifting policy interpretations can have tangible downstream effects on biomedical research, from delayed project initiation to the loss of funding continuity for critical studies addressing public health challenges. This capability is particularly relevant for proposals in sensitive domains—such as social determinants of health, behavioral medicine, and community-based research—that are most vulnerable to evolving policy restrictions. By proactively identifying and mitigating noncompliant language, GrantCheck helps preserve the operational continuity of research programs across a wide spectrum of domains—including infectious disease surveillance, cancer prevention, precision medicine, behavioral health, and health equity. This is particularly important for proposals that engage underserved or marginalized populations, where delayed or rejected applications can exacerbate existing disparities in research representation and access to innovation.

Furthermore, ensuring compliant grant communication supports institutional stability by protecting revenue streams that maintain clinical research infrastructure, data registries, and translational science programs. In large health systems, even short-term disruptions in funding can slow recruitment, delay protocol activation, and diminish capacity to generate evidence that informs care delivery. GrantCheck thus functions as an upstream enabler of public health resilience—helping academic medical centers maintain continuity in studies that contribute to disease prevention, health system innovation, and evidence-based policymaking.

At a broader societal level, integrating AI-driven compliance tools into research administration can strengthen national readiness for future public health emergencies. During crises, such as pandemics, when timely funding and cross-sector collaboration are essential, reducing administrative friction in the grant process becomes critical for mobilizing rapid scientific response. By maintaining the integrity and inclusivity of research language under evolving policies, GrantCheck helps ensure that vital investigations—especially those addressing social determinants of health and population-level interventions—can proceed without unnecessary delay or topic avoidance.

Finally, the responsible, privacy-preserving design of GrantCheck offers a model for scalable digital infrastructure that reinforces public trust in AI-assisted health research workflows. By aligning institutional compliance with ethical transparency and investigator autonomy, this approach not only reduces administrative burden but also strengthens the long-term sustainability and fairness of the biomedical research enterprise. Together, these features position GrantCheck as an enabling technology that links policy compliance to the broader mission of improving health outcomes, advancing equity, and maintaining scientific innovation.

### Conclusions

GrantCheck demonstrates the feasibility of an AI-assisted compliance tool that helps researchers align grant narratives with evolving policy requirements while preserving scientific integrity. Its secure, hybrid regex–LLM architecture and institutional deployment model offer a replicable framework for responsible AI adoption in academic research settings. Future work will expand validation across institutions, integrate stronger assurance benchmarking, and explore multiagent architectures to enhance contextual sensitivity, scalability, and policy adaptability.

## Appendix

Multimedia Appendix 1 System usability survey.

Multimedia Appendix 2 Survey Data.

## Abbreviations

AI: artificial intelligence
AWS: Amazon Web Services
IRB: Institutional Review Board
LLM: large language model
NLP: natural language processing
SSO: single sign-on
SUS: System Usability Scale
VPC: virtual private cloud

## Data Availability

The deidentified survey data generated and analyzed during this study are available in Multimedia Appendix 2.
