# Supplementary material for: GrantCheck—an AI Solution for Guiding Grant Language to New Policy Requirements: Development Study
Source: JMIR Form Res. 2025 Nov 27;9:e79038. doi: 10.2196/79038 (PMC12699247; doi:10.2196/79038)
Supplement: Multimedia Appendix 1 [file formative_v9i1e79038_app1.docx]

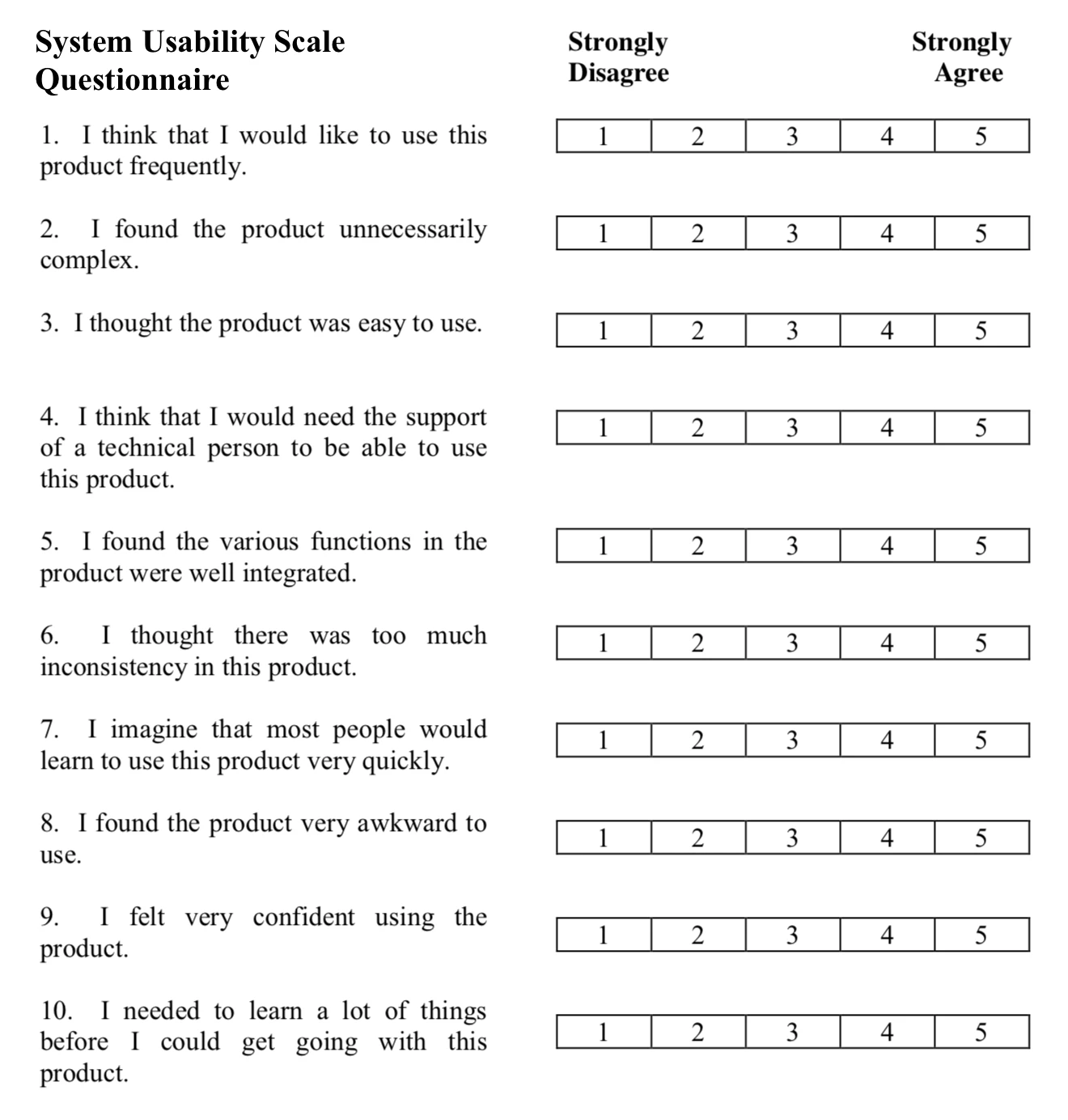


**Appendix 1. System usability survey.** This figure displays the standardized System Usability Scale questionnaire used to evaluate GrantCheck. It includes ten statements rated on a five-point Likert scale from “Strongly Disagree” to “Strongly Agree,” covering perceived usability, complexity, confidence, and integration.
